# Retrospective analysis of canine monocytic ehrlichiosis in Thailand with emphasis on hematological and ultrasonographic changes

**DOI:** 10.14202/vetworld.2022.1-9

**Published:** 2022-01-05

**Authors:** Kris Angkanaporn, Jidapha Sanguanwai, Taratip O. Baiyokvichit, Pichamon Vorrachotvarittorn, Montana Wongsompong, Woraporn Sukhumavasi

**Affiliations:** 1 Department of Veterinary Physiology, Faculty of Veterinary Science, Chulalongkorn University, Bangkok 10330, Thailand; 2 Faculty of Veterinary Science, Chulalongkorn University, Bangkok 10330, Thailand; 3 Parasitology Unit, Department of Veterinary Pathology, Faculty of Veterinary Science, Chulalongkorn University, Bangkok 10330, Thailand.

**Keywords:** dog, ehrlichiosis, hematology, monocyte, ultrasound

## Abstract

**Background and Aim:**

Canine monocytic ehrlichiosis (CME) is a tropical endemic tick-borne disease that causes fatality or chronic infection involving many organs in dogs. This study aimed to examine the prevalence, risk factors, and hematological and ultrasonographic changes in the liver, gallbladder, kidneys, and spleen following CME infection.

**Materials and Methods:**

This retrospective study used 30,269 samples collected from dogs at the hematology section of the pathology unit of a university veterinary hospital and 35 samples collected from dogs at the diagnostic imaging unit. CME was determined using the buffy coat smear method. Data were analyzed using descriptive statistics and odds ratios.

**Results:**

The data revealed that the average yearly prevalence of CME was 1.32%. Risk factors contributing to CME infection were a tick on the body during physical examination, lack of ectoparasite control, and outdoor living. All 148 dogs with CME infection had low platelet counts. The percentages of CME-infected dogs with elevated serum alanine aminotransferase, alkaline phosphatase, and both enzymes above the normal range were 33.6%, 65.9%, and 29.8%, respectively. The rates for elevated serum levels of blood urea nitrogen, creatinine, and both compounds were 33.1%, 19.1%, and 17.3%, respectively. The most common ultrasonographic changes were liver abnormalities (hyperechogenicity or hypoechogenicity, hepatomegaly, and hypoechoic nodules), hyperechogenicity of the kidneys, and an enlarged spleen. These ultrasonographic changes were consistent with the hematology results, which showed a greater elevation of serum liver enzyme levels than renal enzymes.

**Conclusion:**

Ultrasonographic changes during CME infection and after treatment with doxycycline can help to monitor and identify persistent pathological changes in the target organs resulting from immune response to CME.

## Introduction

Canine monocytic ehrlichiosis (CME) is a tick-borne, endemic disease found worldwide that causes animals in the *Canidae* family to become sick and die. The disease is caused by rickettsial bacteria, namely, *Ehrlichia canis*, transmitted by *Rhipicephalus sanguineus* (brown dog tick). CME is widely distributed in tropical, Mediterranean, and subtropical climates, including Europe [1,2], the United States [3], Costa Rica [4], Brazil [5], and Asia [6-8]. In Thailand, the reported prevalence of *E. canis* identified using polymerase chain reaction (PCR) in all parts of the country ranges from 7.6% to 38.3% [9-13]. Transstadial transmission occurs in all tick stages, and infection can result while feeding on infected dogs. On 4-7 days after infection (dai), the dog’s immune system develops immunoglobulin M and immunoglobulin A antisera, and immunoglobulin G antisera can be detected at 15 dai [14]. Following the 8-20 days incubation period, CME infection progresses through three typical phases; acute, subclinical, and chronic. In the acute phase, which lasts for 3-5 weeks, the clinical symptoms of fever, anorexia, ocular discharge, mucosal and skin petechiae, epistaxis, pale mucous membrane, hemorrhagic tendencies, depression, lymphadenopathy, and neurological signs (from meningitis) are present [15]. The major hematological changes are interstitial nephritis and glomerulonephritis [16], whereas pathological changes occur in the corticomedullary junction, causing a contracted kidney [17]. Hyperechogenicity may be present with an enlarged liver, spleen, gallbladder, and ascites [18]. Some dogs may recover after the subclinical phase, whereas others may progress to the chronic phase where severe pancytopenia typically occurs from bone marrow hypoplasia and leads to severe leukopenia, anemia, and thrombocytopenia with a high risk of mortality [15]. In severe cases, dogs with poor antibiotic response may die from massive hemorrhage, severe debilitation, and/or secondary infection [15]. During the chronic phase, pathological lesions occur in the kidney because of immune complex accumulation in the glomerulus that stimulates inflammation, followed by the destruction of cells and tissues in the surrounding area, leading to elevated serum blood urea nitrogen (BUN) and creatinine levels. There are also lymphocyte and plasma cell infiltration into the liver and kidney parenchyma [15], and moderate increases in serum levels of the liver alanine aminotransferase (ALT) and alkaline phosphatase (ALP) due to hepatocyte damage [15,19].

With the standard doxycycline protocol treatment [14], some dogs may not fully recuperate from symptoms related to the immune response, especially damage to the principal organs involved (liver, kidney, and spleen). These lasting effects may be missed by veterinarians that do not provide systematic follow-up after doxycycline treatment. Sarma *et al*. [18] studied pathological changes in the liver and spleen of 101 dogs positive for infection with tick-borne blood parasites and found that ultrasound and hematological changes can serve as a useful indicator of the damage status of internal organs after infestation with blood parasites.

Although there are several reports [9-12] on the prevalence of CME in Thailand, there is scarce research on the relationship between CME and changes in ultrasound images of dogs during or after treatment. Thus, the present study aimed to investigate the retrospective prevalence of CME in dogs and examine changes in blood parameters and organs (liver and kidney) of infected dogs as revealed by ultrasound images.

## Materials and Methods

### Ethical approval and informed consent

Because of the retrospective nature of this study and the use of diagnostic data collected as a part of routine clinical procedures, the need for ethical approval was waived. All dog owners completed a consent form giving permission to utilize the data (including ultrasound images) for clinical research.

### Study period and location

This study was divided into two parts. part 1 was performed at the Small Animal Teaching Hospital, Faculty of Veterinary Science, Chulalongkorn University from September 2016 to August 2017. Part 2 of this study was performed on dogs that were admitted and underwent examinations at the Hematology Section, Pathology Unit and Imaging Diagnostic Unit of the same Small Animal Hospital from January 2017 to September 2018.

### Study design and analysis

A retrospective, randomized study was performed based on hematological and medical records. We divided the study into two parts in order to study the prevalence of CME based on the yearly data as well as the factors involved as in part 1. In part 2, the retrospective case-control comparison of dogs that had data on ultrasonographical and blood analysis was examined.

#### Study part 1

We identified a group of CME-positive dogs, defined by the presence of the morulae of *Ehrlichia* spp. in the buffy coat smear assay and results of the Canine SNAP^®^ 4Dx^®^ test kit (IDEXX Laboratories, Inc., Westbrook, ME, USA). The prevalence of CME during the study period was determined. To understand factors influencing CME risk, we analyzed the following data: Signalment data; historical records; complete blood count (CBC) data, including platelet count; and blood chemistry data, including serum levels of ALT, ALP, BUN, and creatinine. Duplicate data were removed before analysis.

Next, based on the serum platelet count and blood chemistry data (ALT, ALP, BUN, and creatinine), the dogs that were *E*. *canis* positive were grouped as below, within, or above the normal range for these measures. Moreover, the dogs that were *E*. *canis* positive were analyzed for (i) the presence of ticks on the body during physical examination, (ii) use of an ectoparasite control program, and (iii) daily indoor or outdoor living. Ectoparasite control was defined as consistent and routine control using approved products. For daily indoor or outdoor living, only dogs that spent 100% of their time indoors were considered indoor living dogs. For comparison, healthy dogs were randomly chosen from the historical data to serve as a control group. The inclusion criteria were dogs with no severe diseases or CME. The numbers of control dogs were similar to those with CME (150, 57, and 40 dogs for small, medium, and large breeds, respectively) (Table-1). The odds ratio (OR) was computed for comparisons between CME and control (healthy) groups.

**Table 1:** The OR for *E. canis* infection (buffy coat smear method) compared to the control group in dogs of different weight classes (Data from September 2016 to August 2017).

	Body weight (kg)	Tick			Ectoparasite control			Living			n
		
		Found	Not found	OR*	Control	Not control	OR*	Indoor	Outdoor	OR*	
Control	<16	4	146	-	93	57	-	133	17	-	150
	16-25	2	55	-	42	15	-	20	37	-	57
	>25	4	36	-	35	5	-	24	16	-	40
Positive E. canis**	<16	27	123	8.0	68	82	2.0	87	63	5.7	150
	16-25	18	39	12.7	20	37	5.2	17	40	1.3	57
	>25	10	30	3.0	26	14	3.8	20	20	1.5	40

* Odds ratio, control group compared to the positive group. n=Numbers of dogs in each weight group. The total N for the positive *E. canis* group (247 dogs) was less than the data in Tables 1 and 2 due to incomplete history on various factors examined. The total n for the control group (247 dogs) was randomly selected to match with the positive group according to weights.

** Using the buffy coat smear method. *E. canis*=*Ehrlichia* *canis*

#### Study part 2

From the data, dogs were selected using non-probability or non-random sample selection. The inclusion criteria did not restrict the gender or breed. Nevertheless, the dogs must not be older than seniors because geriatric dogs may show age-related pathophysiological changes in the ultrasound appearance of the liver or kidneys unrelated to *E. canis* infection. We also excluded dogs with a history of severe diseases, including heart, liver, kidney, cancer, and immune system diseases.

Data were divided into a control group and a study group. The control group comprised 16 dogs with normal abdominal ultrasound results for their internal organs. The study group comprised 19 dogs positive for *E. canis* infection that showed abnormal abdominal ultrasound results in at least one of the periods before, during, or after infection (treatment with doxycycline at 10 mg/kg/day for 28 days).

We analyzed the effect of *E. canis* infection on hematological changes, including CBC and serum data, including the levels of the liver (ALT and ALP) and renal (BUN and creatinine) enzymes. The blood parameters were analyzed before, during, and after (treatment with doxycycline at 10 mg/kg/day for 28 days) *E. canis* detection. Hematological results were categorized as normal and abnormal when compared with normal reference values [20]. The effects of *E. canis* infection on changes in the serum levels of platelets and liver and renal enzymes were analyzed using descriptive statistics, with some constraint on the missing data in the historical records.

The effect of *E. canis* infection in both groups (control and study groups) was analyzed in relation to ultrasonography changes in the three periods, as described above, for the liver, spleen, kidneys, and gallbladder, as these are the organs typically affected by *E. canis* infection.

### Statistical analysis

Descriptive statistics were used to analyze and compare all parameters in Parts 1 and 2. The prevalence of *E. canis* infection was reported as the mean value calculated on a yearly basis. In Part 1, the Odds ratio (OR) was used to measure the association between the control and *E. Canis*-positive groups in terms of (i) the presence of ticks on the body during physical examination, (ii) ectoparasite control program, and (iii) daily indoor or outdoor living. Statistical analysis was performed using Sigmastat (Systat Software, San Jose, CA, USA). p<0.05 was considered to be statistically significant.

## Results

### Part 1

All infected dogs had platelet counts below the normal range. The percentages of infected dogs with elevated serum liver enzymes ALT, ALP, and both above the normal range [20] were 33.6%, 65.9%, and 29.8%, respectively; the rates of elevated kidney markers BUN, creatinine, and both were 33.1%, 19.8%, and 17.3%, respectively (Table-2) [20].

**Table 2:** Percentage of dogs that found *Ehrlichia canis* by buffy coat smear method with platelets counts and blood chemistry was separated into three groups; below, within, and above from normal range of platelet counts, blood chemistry record including SNAP4DX tested from September 2016 to August 2017.

Parameter	Normal range^1^	Below (%)	Within (%)	Above (%)
Platelets	(211,000-600,000)	100	0	0
ALT	(10-109)	0.3	66.4	33.6
ALP	(1-114)	0	31.1	65.9
ALT and ALP	−	0	29.8	29.8
BUN	(8-28)	1.1	65.9	33.0
Creatinine	(0.5-1.7)	3.0	77.2	19.8
BUN and creatinine	−	0	58.5	17.3
SNAP4DX positive number tested/total number (percentage)		415/626 (66.3%)		

1 Reference normal range [20]. *E. canis=Ehrlichia canis*

**Figure-1:**
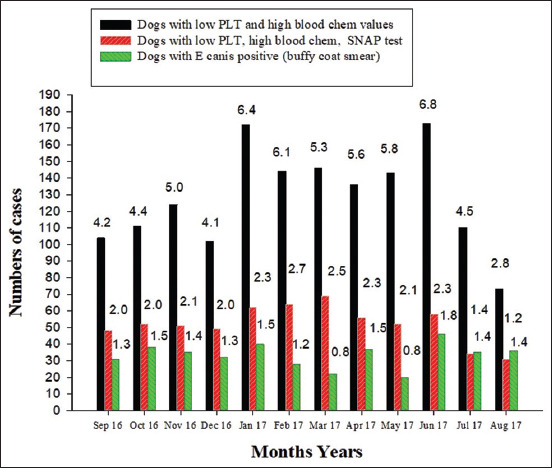
Numbers of dogs with low platelet, high serum chemistry profiles (black bar), and SNAP4DX testes (red bar), including those with *Ehrlichia canis* positive using buffy coat smear (green bar) in the experimental period of the study (from September 2016 to August 2017). The value n the top of each bar represents the percentage of number of case compared to number of total dogs having blood tests.

Data from the retrospective study identified 400 of the total 30,269 dogs with a positive *E. canis* test. The prevalence of *E. canis* infection was 1.32% with a range of 0.8-1.8% each month. The percentage of dogs with low platelet and high serum chemistry profile ranged from 2.8% to 6.8% each month with an average of 5.1% (Figure-1). Approximately two-thirds (66.3%) of the SNAP4Dx tests were positive (Table-2).

Table-1 summarizes the OR of various factors affecting *E*. *canis* infection in the different groups of dogs, according to body weight. The occurrence of ticks on the body during physical examination was associated with 8.0, 12.7, and 3.0-fold higher rate of CME in dogs weighing <16, 16-25, and >25 kg, respectively. In the ectoparasite control analysis, the “no control” regime was associated with a 2.0-, 5.2-, and 3.8-fold higher risk of CME in the three weight groups, respectively, when compared with the “control” regime. Finally, outdoor dogs had 5.7, 1.3, and 1.5-fold greater risk of CME (concerning the weight groups) when compared with indoor dogs.

### Part 2

#### Hematological and blood data changes in E. canis infected dogs

Data from 19 dogs in the study group were included in this analysis. The age was known in all cases, and the mean group age was 7 years (range of 3 months-11 years). In terms of sex, 47.4% (9 of 19) were female and 52.6% (10 of 19) were male. The two groups included both entire and neutered animals. There were 10, 6, and 3 cases of small, medium, and large breeds, respectively.

#### Hematology

The CME-positive dogs were analyzed in the phase before the presence of *E. canis* (Table-3). The platelet concentration data from 15 cases revealed 4 dogs (26.7%) with normal levels and 11 (73.3%) with decreased platelet concentrations. In the presence of *E. canis*, all 19 cases (100%) showed a markedly reduced platelet concentration (38,240±23,369/mL). After treatment with doxycycline, 4/11 dogs (36.4%) with available data still had a decreased platelet level (90,000±66,878/mL).

**Table 3:** Serum platelet levels in *E. Canis*-infected dogs (buffy coat smear method) at three different infection phases.

Phase	Normal platelet count	Decreased platelet count	No data
Before presence of *E. canis*	4 (295,000±73,289)	11 (83,818±53,842)	4
During presence of *E. canis*	0	19 (38,240±23,369)	0
After treatment with doxycycline	7 (432,000±231,062)	4 (90,000±66,798)	8

*Number in parentheses represents the mean±SD*. E. canis=Ehrlichia canis*

#### Serum chemistry profiles

Liver markers (ALT and ALP)

Of the 19 *E. canis* infected dogs, 13 (68.4%) had elevated serum ALT levels (647±1083 IU/L). The levels remained elevated, albeit at a lower magnitude, after treatment with doxycycline in 7/11 (63.6%) cases (422±326 IU/L), whereas 4/11 (36.4%) cases had reverted to normal levels of ALT (Table-4). Similarly, an increased serum ALP level was observed in the presence of *E. canis* in 17/19 (89.5%) cases (698±449 IU/L). The levels remained elevated in 9/11 (81.2%) cases after treatment with doxycycline, but at an even higher extent (948±968 IU/L), with only 2/11 (18.2%) cases returning to within the normal range (Table-4). Hence, the serum levels of ALP were in accordance with those for ALT and consistent with liver damage.

**Table 4:** Numbers of dogs with normal or increased serum levels of liver markers (ALP and ALT) in *E. Canis*-infected cases (buffy coat smear method) during different infection phases.

Phase	Normal ALT	Increased ALT	No data	Normal ALP	Increased ALP	No data
Before *E. canis*	9 (40±28)	5 (489±372)	5	4 (48±24)	9 (720±451)	6
During *E. canis*	6 (29±25)	13 (647±1,083)	0	2 (53±18)	17 (698±449)	0
After treatment with doxycycline	4 (53±42)	7 (422±326)	8	2 (161±36)	9 (948±968)	8

*Number in parentheses represents the mean±SD. *E. canis=Ehrlichia canis*, ALT = Alanine aminotransferase,

ALP = Alkaline phosphatase

Kidney markers (BUN and creatinine)

In the presence of detectable *E. canis*, increased serum levels of BUN (52±12 mg%) were evident in 5/18 (27.8%) cases. Although normal serum levels of BUN were observed in 8/11 (72.7%) cases after treatment with doxycycline, 3/11 cases (27.3%) still showed increased BUN levels (50±5 mg%) (Table-5). Normal serum levels of creatinine were found in most cases (11/14, 78.6%) before detectable infection but increased (1.7±0.3 mg%) in the presence of *E. canis* in 15/18 (83.3%) cases. After treatment, creatinine levels were within the normal limits in all cases (11/11, 100%; Table-5).

**Table 5:** Numbers of dogs with normal or increased serum levels of kidney markers (BUN and creatinine) in *E. Canis*-infected dogs (buffy coat smear method) during different infection phases.

Phase	Normal BUN	Increased BUN	No data	Normal creatinine	Increased creatinine	No data
Before *E. canis*	11 (20±8)	2 (40±8)	6	11 (0.9±0.2)	3 (1.7±0.3)	5
During *E. canis*	13 (15±3)	5 (52±12)	1	15 (0.7±0.2)	13 (0.95±0.33)	1
After treatment with doxycycline	8 (22±7)	3 (50±5)	8	11 (0.7±0.2)	0	8

*Number in parentheses represents the mean±SD. *E. canis=Ehrlichia canis*, BUN=Blood urea nitrogen

#### Effects of E. canis infection on ultrasound appearance of the liver, gallbladder, kidneys, and spleen

Abdominal ultrasonographic examination results of the liver, gallbladder, and kidneys in all 16 cases in the control group were found to be normal. The liver showed a normal sharp border with a smooth margin, good location, contours with a homogeneous echotexture, normal appearance of the intrahepatic portal veins, uniform hypoechoic liver parenchyma related to the spleen, and falciform fat with isoechoic to the right renal cortex. Additional observations included a normal gallbladder wall thickness and anechoic bile content; a normal appearance of both kidneys in terms of size, shape, location, contour, and echotexture; normal renal cortex echogenicity; a well-defined corticomedullary junction; and a normal renal pelvis and smooth renal capsule.

**Figure-2:**
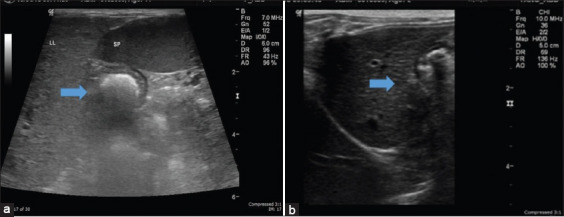
Hyperechoic parenchyma of the liver compared with the spleen in the (a) *Ehrlichia canis-*infected and (b) control groups. The arrow indicates the difference between infected and control dogs.

Ultrasonographic changes in the liver in the presence of *E. canis* were noted in all 13 infected cases. Hyperechogenicity of the liver was observed in 7 (53.8%) cases, whereas 4 (30.8%) cases revealed hypoechoic hepatic parenchyma. Hepatomegaly was observed in 10 cases (76.9%), as shown in Table-6 and Figure-2. After treatment with doxycycline, 4 (30.8%) and 3 (23.1%) cases still showed hyperechogenicity and hepatomegaly, respectively, of the liver (Table-6).

**Table 6:** Ultrasonographic changes in the liver, gallbladder, kidney, and spleen during detectable *E. canis* infection (buffy coat smear) and after doxycycline treatment (10 mg/kg BW, 28 day).

Organ	Ultrasonographic changes	Control group (n=16)	With *E. canis* (n=13)	Post-infection (n=6)
Liver	Hyperechogenicity	0	7	4
	Hypoechogenicity	0	4	0
	Hepatomegaly	0	10	3
	Hypoechoic nodule	0	2	1
Gallbladder	Gallbladder distention	0	2	4
Kidney	Hyperechogenicity	0	3	2
Spleen	Splenomegaly	0	10	4
Hypoechogenicity		0	8	3

*E. canis=Ehrlichia canis*

#### Ultrasonographic changes in the gallbladder, kidneys, and spleen

**Figure-3:**
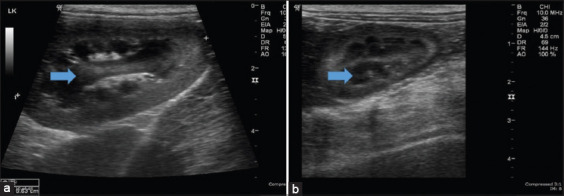
Ultrasound images of the kidneys (a) in *Ehrlichia canis-*infected and (b) control groups. The arrow indicates the difference between infected and control dogs.

In the presence of *E. canis*, 2/13 (15.4%) cases showed gallbladder distention. This was still evident in 4/6 (66.7%) cases after treatment with doxycycline (Table-6). For the kidneys, hyperechogenicity was evident in 3/13 (23.1%) cases in the presence of *E. canis* and persisted in 2/6 (33.3%) cases after treatment with doxycycline (Table-6 and Figure-3).

Splenomegaly was found in 10/13 (76.9%) cases infected with *E. canis*, whereas 8/13 (61.5%) cases showed hypoechoic spleens (Table-6). After treatment with doxycycline, there were still 4/6 (66.7%) and 3/6 (50%) cases of splenomegaly and hypoechogenicity, respectively (Table-6).

## Discussion

In Thailand, there is no seasonal difference in the prevalence of *E. canis* infections. The yearly prevalence rate of *E. canis* was found to be 1.32% in the present study using the buffy coat smear method. There are several alternative techniques to diagnose CME apart from buffy coat smears, such as SNAP4D_X_, PCR, and immunofluorescence assay [14,15]. Nevertheless, the buffy coat smear method remains the most common method for screening *E. canis* infection in clinics because of its convenience and relatively low cost. Nevertheless, there are some limitations of the buffy coat smear method, which has a sensitivity and specificity of 16.1% (confidence interval [CI]=10.7–23.6%) and 89.4% (CI=85.0–92.6%), respectively, resulting in a high chance of false-negative results but a low rate of false positives. This could be the reason for the low prevalence of *E. canis* infection in our study. A composite study in India reported that the overall prevalence rates of ehrlichiosis by microscopic examination, commercial dot-ELISA, and nested PCR assay were 1.3%, 19.1%, and 5.8%, respectively [21]. The rate determined by microscopic examination is similar to that reported for Thailand, although the occurrence of CME using the PCR test was higher than that in India [9-13,22]. The sensitivity of an *E. canis* test also depends on the stage of infection at the time of sampling. In the acute phase, there is more opportunity to find infected leukocytes in the blood smear because of the higher degree of parasitemia. However, in the subclinical and chronic phases, the chances of finding infected leukocytes decrease, which can lead to false negatives. However, the probability of *E. canis* detection by specific antibodies, such as through ELISA, increases in the chronic phase because the secondary immune system (and thus immunoglobulin levels) requires time to respond [23].

In this study, all *E. Canis*-positive dogs showed a serum platelet count below the normal range. The magnitude of reduced platelet count has been suggested as a useful screening test for CME in endemic regions. Although only 1/71 (1.4%) of non-thrombocytopenic dogs (platelet count > 200,000/mL) were found to be positive for *E. canis* DNA (16S rRNA) through PCR, 13/62 (21%) dogs with platelet counts of 100,000-200,000/mL and 53/84 (63.1%) dogs with platelet counts of <100,000/mL were PCR positive for *E. canis* [24]. This highlights the importance of evaluating true platelet counts in dogs suspected to have *E. canis* infection, since most infected dogs have thrombocytopenia as the main clinical sign. Thrombocytopenia in CME is attributed to various mechanisms across the different stages of the disease. In the acute stage, the cause is increased platelet consumption because of vasculitis, splenic sequestration of platelets, and immunologic destruction [15,25]. In addition, infected dogs show a significantly decreased platelet life span and increased mean platelet volume [25]. Platelet destruction by the immune response may be associated with the serum platelet bindable antiplatelet antibody, which is produced 17 dai. During the severe chronic phase, platelet production is decreased because of bone marrow hypoplasia, which can lead to pancytopenia [15].

CME can occur in dogs of any age or breed. Higher seropositive levels were found in male dogs compared to female ones, which has been explained by male dogs’ higher exposure to vectors due to their behavioral characteristics [26]. Other factors associated with exposure to CME agents are the dog’s habitat, contact with other dogs, and the presence of ticks. Dogs that have contact with other dogs and dogs parasitized by the ticks of *R. sanguineus*, which are the vector of *E. canis*, showed a higher likelihood of exposure [3,6,13]. Since *R. sanguineus* is a three-host tick species, it must complete its life cycle on the ground. Outdoor living dogs are, therefore, expected to be at higher risk of CME than indoor living dogs [27], as demonstrated in this study.

Similarly, dogs with ticks on their body are more susceptible to CME infection. Hence, one effective way to prevent *E. canis* infection in dogs is tick control [26]. However, even with ectoparasite control, some dogs still develop CME. This can be explained by how effectively the owners control ticks because only some owners understand that preventing ticks on the dog’s body can prevent CME. To improve owners’ knowledge about ticks, education should focus not only on preventing dogs from becoming infested with ticks but also on measures for environmental control of ticks [28]. The brown dog tick is most abundant during the hot and humid periods of the year, particularly in Thailand [27]. The prevalence and epidemiology of ticks also depend on geographical locations [16,27]. The prevalence of ticks is as high as 80% in some areas, such as in Northeast Thailand. A high temperature (25-35°C) supports tick development and the success of laying, hatching eggs, and larval and nymphal molting, which may explain why ticks are more prevalent in the summer in several countries [27]. However, in Thailand, the weather is broadly similar to a hot and humid climate almost all year, suitable for ticks to mate and develop.

In Part 2 of the study, the platelet concentrations in the period before detection of *E. canis* infection were still normal in some of the dogs. So they appeared uninfected in the first phase of infection. Similar to our results, the number of platelets was previously reported to be normal in the first 2 weeks after CME infection and then decreased significantly from the 3^rd^ to 5^th^ weeks [29]. In the present study, thrombocytopenia was detected in other dogs in the period before infection, which may be because of a false-negative blood smear test result or because some dogs may develop thrombocytopenia because of other causes. All 19 dogs in our study with detectable *E. canis* infection had thrombocytopenia. This result is consistent with Bulla *et al*. [24], who reported that dogs infected with *E. canis* in the acute and subclinical phases had mild thrombocytopenia but showed severe thrombocytopenia in the chronic stage. Although platelet levels returned to normal in 7/11 dogs in the post-treatment period (after doxycycline treatment for 28 days), 4/11 dogs still showed markedly lower platelet levels than normal [20]. This result is in accordance with the study of Villaescusa *et al*. [30], who treated CME-infected dogs with doxycycline at a dose of 10 mg/kg/day for 28 days and found that the platelet counts increased to the normal level 180 days after treatment. When doxycycline was administered to control group dogs, they also showed increased levels of platelets. Doxycycline may increase platelet counts; however, the mechanism is unknown. It is common and confirmed by our study that thrombocytopenia in some dogs persists after treatment to eradicate *E. canis* infection. Hence, platelet counts should be examined routinely after treatment with doxycycline.

In the present study, we found that the rates of serum hepatic enzymes (ALT and ALP) above the normal range were 33.6% and 65.9%, respectively, in infected dogs, whereas increased kidney enzymes (BUN and creatinine) were present in 33.1% and 19.8%, respectively, of dogs. Taken together, these results suggest liver and kidney damage. The liver histopathology in infected dogs demonstrated infiltration of plasma cells, lymphocytes, and macrophage cells around the centrilobular veins and in the portal triads. Centrilobular fatty degeneration and perivascular and portal plasmacytosis were previously reported in naturally infected, chronic case of CME infected dogs [31]. In addition, dark blue cytoplasmic inclusions, which are consistent with Ehrlichia morulae, have been observed in lymphocytes and macrophages [32]. Renal protein decreases have also been reported in *E. Canis*-infected dogs, resulting in the increased urinary protein to creatinine ratio (average ratio=8.6) during the 3^rd^ and 4^th^ weeks after infection, which decreased to <0.5 by 6 weeks after infection. The hypoalbuminemia associated with acute *E. canis* infection may primarily contribute to the increased loss of renal protein rather than decreased hepatic synthesis [33]. The renal lesions in acutely infected dogs showed perivenular and interstitial infiltrate of lymphocytes and plasma cells localized principally to the renal cortex [33]. Glomerular lesions were minimal to absent. These results suggest that a minimal change in glomerulopathy can cause proteinuria without histological evidence of renal disease rather than immune complex glomerulonephritis [33]. The results of this study show that veterinarians should recognize the importance of monitoring clinical signs, hematology (e.g., hematocrit), platelet counts, and serum chemistry profiles particularly ALT, ALP, BUN, and creatinine levels to identify recurrent or resistant CME. Increased serum levels of liver enzymes were found in infected dogs both before and after treatment with doxycycline in this study. There was no significant difference between serum liver enzymes changes, such as ALT, in uninfected dogs and those treated with doxycycline [30]. The serum renal enzyme levels in some dogs with *E. canis* detected in the bloodstream were higher than normal levels. In dogs treated with doxycycline, the serum levels of renal enzymes decreased slightly back to normal values, which were likely because of the action of tetracycline at nephron sites in the kidneys [30].

In the present study, abdominal ultrasonography of the CME-infected dogs revealed hypoechogenicity of the liver, gallbladder distension, and hepatomegaly. Notably, Sarma *et al*. [34] reported the same findings. Mylonakis *et al*. [31] reported enlarged and diffusely hypoechoic liver in *E. canis* infected dogs, whereas severe hepatitis induced by *E. canis* has been documented as a portal infiltration of lymphocytes, plasma cells, and macrophages, resulting in a pronounced distortion of the surrounding acinar architecture [34]. This is associated with ultrasonographic changes in the liver that revealed decreased liver parenchyma echogenicity.

In the case of tick-borne intracellular diseases, hepatomegaly may be due to passive congestion, reticuloendothelial hyperplasia, or infiltrative diseases mediated through cytokines [35]. The sonographic changes observed in the gallbladder included distention with the presence of sludge/clear bile, which may be due to anorexia [35]. Hyperechogenicity of the liver was also observed in the present study, which has been previously reported in chronic CME infections [36]. Sarma *et al*. [18] also reported hyperechogenicity of the liver, gallbladder distention, and hepatosplenomegaly concomitant with tick-borne disease. Splenomegaly was also observed in our study, which is consistent with the findings reported by Sarma *et al*. [34]. Multiplication of *E. canis* within circulating mononuclear cells and mononuclear phagocytic tissues of the spleen has been shown to result in hepatomegaly [32].

The kidney showed a hyperechoic echotexture compared with the spleen in the present study, which is presumably related to the deposition of immune complexes in the kidneys that trigger glomerulonephritis and predispose dogs to proteinuria [15]. Interstitial nephritis in dogs with CME was also observed, which is associated with lymphocyte infiltration and suggests that these cells may also play an important role in the immunopathogenesis of renal lesions [37]. Although doxycycline can successfully clear *E. canis* infection when administered for 4 weeks, another study [38] reported persisting abnormalities of the liver and kidney through ultrasonography after treatment. Mcclure *et al*. [39] reported that the treatment of dogs with acute or subclinical CME with doxycycline for 28 days resulted in them becoming PCR negative for *E. canis* along with improved clinical parameters. Nevertheless, in the chronic CME cases in this study, there were still abnormalities in hematology, serum chemistry profiles, and ultrasonographic changes in the liver, kidney, and spleen after treatment with doxycycline for 28 days.

Given the constraints of this study, it was not possible to examine ultrasonography data before *E. canis* detection, which limits the ability to explain changes before, during, and after CME infection. Nevertheless, the present study has demonstrated the value of ultrasound examination of the liver, kidneys, and spleen, as these organs are susceptible to change during CME infection and after doxycycline treatment. Veterinarians should be aware of the potential need to treat liver and kidney disorders, especially after 28 days of doxycycline treatment.

## Conclusion

CME induces liver and renal pathological changes, leading to increased serum ALT, ALP, BUN, and creatinine levels. Despite treatment with doxycycline at 10 mg/kg/day for 28 days, a persistent increase in serum levels of liver and kidney enzymes was observed in some dogs. Ultrasonographic changes during and after doxycycline treatment can help monitor and indicate persistent pathological changes in the target organs.

## Authors’ Contributions

KA: Designed the study, statistical analysis, and manuscript writing and editing. JS and MW: Assisted in the ultrasonography, collected and tabulated clinical data in part 2. TOB and PV: Collected and tabulated data in part 1. WS: Assisted in the manuscript writing. All authors read and approved the final manuscript.

## References

[1] René-Martellet M, Lebert I, Chêne J, Massot R, Leon M, Leal A, Badavelli S, Chalvet-Monfray K, Ducrot C, Abrial D, Chabanne L, Halos L (2015). Diagnosis and incidence risk of clinical canine monocytic ehrlichiosis under field conditions in Southern Europe. Parasit. Vectors.

[2] Bilgin B.H, Kirli P.G, Murat H, Turin K (2019). A retrospective epidemiological study:the prevalence of *Ehrlichia canis* and *Babesia volgeli* in dogs in the Algean region of Turkey. Acta. Vet. Beograd.

[3] Gettings J.R, Self S.C.W, McMahan C.S, Brown D.A, Nordone S.K, Yabsley M.J (2020). Local and regional temporal trends (2013-2019) of canine *Ehrlichia* spp. seroprevalence in the USA. Parasit. Vectors.

[4] Barrantes-González A.V, Jiménez-Rocha A.E, Romero-Zuniga J.J, Dolz G (2016). Serology, molecular detection and risk factors of *Ehrlichia canis* infection in dogs in Costa Rica. Ticks Tick Borne Dis.

[5] Paulino P.G, Pires M.S, da Silva C.B, Peckle M, da Costa R.L, Vitari G.V, Vilela J.A.R, de Abreu A.P.M, Massard C.L, Santos H.A (2018). Epidemiology of *Ehrlichia canis* in healthy dogs from the Southeastern region of the state of Rio de Janeiro, Brazil. Prev. Vet. Med.

[6] Rani P.A.M, Irwin P.J, Coleman G.T, Gatne M, Traub R.J (2011). A survey of canine tick-borne diseases in India. Parasit. Vectors.

[7] Ansari-Mood M, Khoshnegah J, Mohri M, Rajaei S.M (2015). Seroprevalence and risk factors of *Ehrlichia canis* infection among companion dogs of Mashhad, North East of Iran, 2009-2010. J. Arthropod Borne Dis.

[8] Nazari M, Lim S.Y, Watanabe M, Sharma R.S.K, Cheng N. A. B, Watanabe M (2013). Molecular detection of *Ehrlichia canis* in dogs in Malaysia. PLoS Negl. Trop. Dis.,.

[9] Poolsawat N, Tazawa K, Junsiri W, Watthanadirek A, Srionrod N, Chawengkirttikul R, Anuracpreeda P (2021). Molecular discrimination and genetic diversity of three common tick-borne pathogens in dogs in Thailand. Parasitology.

[10] Piratae S, Senawong P, Chalermchat P, Harnarsa W, Sae-Chue B (2019). Molecular evidence of *Ehrlichia canis* and *Anaplasma platys* and the association of infections with hematological responses in naturally infected dogs in Kalasin, Thailand. Vet. World.

[11] Rucksaken R, Maneeruttanarungroj C, Maswanna T, Sussadee M, Kanbutra P (2019). Comparison of conventional polymerase chain reaction and routine blood smear for the detection of *Babesia canis*, *Hepatozoon canis, Ehrlichia canis,* and *Anaplasma platys* in Buriram province, Thailand. Vet. World.

[12] Lorsirigool A, Pumipuntu N (2020). A retrospective study of dogs infected with *Ehrlichia canis* from 2017-2019 in the Thonburi area of Bangkok province, Thailand. Int. J. Vet. Sci.

[13] Do T, Phoosangwalthong P, Kamyingkird K, Kengradomkij C, Chimnoi W, Inpankaew T (2021). Molecular detection of tick-borne pathogens in stray dogs and *Rhipicephalus sanguineus* sensu lato ticks from Bangkok, Thailand. Pathogens.

[14] Mylonakis M.E, Theodorou K.N (2017). Canine monocytic ehrlichiosis:An update on diagnosis and treatment. Acta. Vet. Beograd.

[15] Waner T, Harrus S (2013). Canine monocytic ehrlichiosis-from pathology to clinical manifestations. Isr. J. Vet. Med.

[16] Oliveira B.C.M, Ferrari E.D, Viol M.A, Andre M.R, Machado R.Z, de Aquino M.C.C, Inacio S.V, Gomes J.F, Guerrero F.D, Bresciani K.D.S (2019). Prevalence of *Ehrlichia canis* (Rickettsiales:Ehrlichieae) DNA in tissues from *Rhipicephalus sanguineus* (Acari:Ixodidae) ticks in areas endemic for canine monocytic ehrlichiosis in Brazil. J. Med. Entomol.

[17] Behera S.K, Hoque M, Sharma K, Saravanan M, Monsang S.W, Mohanta R.K (2012). Abdominal ultrasonographic findings in dogs with canine monocytic ehrlichiosis. Indian Vet. J.

[18] Sarma K, Mondal D.B, Saravanan M, Karunanithy M (2015). Evaluation of haemato-biochemical and oxidative indices in naturally infected concomitant tick borne intracellular disease in dogs. Asia Pac. J. Trop. Dis.

[19] Mylonakis M.E, Harrus S, Breitschwerdt E.B (2019). An update on the treatment of canine monocytic ehrlichiosis (*Ehrlichia canis*). Vet. J.

[20] Latimer K.S (2011). Generating and interpreting test result test validity. Veterinary Laboratory Medicine:Clinical Pathology.

[21] Mittal M, Kundu K, Chakravartid S, Mohapatra J.K, Nehra K, Sinha V.K, Sanjeeth B.S, Churamani C.P, Kumar A (2017). Canine monocytic ehrlichiosis among working dogs of organised kennels in India:A comprehensive analysis of clinico-pathology, serological and molecular epidemiological approach. Prev. Vet. Med.

[22] Nambooppha B, Rittipornlertrak A, Tattiyapong M, Tangtrongsup S, Tiwananthagorn S, Chung Y.T, Sthitmate N (2018). Two different genogroups of *Ehrlichia canis* from dogs in Thailand using immunodominant protein genes. Infect. Genet. Evol.

[23] Guedes P.E.B, Oliveira T.N.D, Carvalho F.S, Carlos R.S.A, Albuquerque G.R, Munhoz A.D, Wenceslau A.A, Silva F.L (2015). Canine ehrlichiosis:Prevalence and epidemiology in Northeast Brazil. Rev. Bras. Parasitol. Vet.

[24] Bulla C, Takahira R.K, Araújo J.P, Trinca L.A, Souza R.L, Wiedmeyer C.E (2004). The relationship between the degree of thrombocytopenia and infection with *Ehrlichia canis* in an endemic area. Vet. Res.

[25] Smith D.R, Ristic M, Huxsoll D.L, Baylor R.A (1975). Platelet kinetics in canine ehrlichiosis:Evidence for increased platelet destruction as the cause of thrombocytopenia. Infect. Immun.

[26] Sainz A, Roura X, Miro G, Estrada-Peña A, Kohn B, Harrus S, Solano-Gallego L (2015). Guideline for veterinary practitioners on canine ehrlichiosis and anaplasmosis in Europe. Parasit. Vectors.

[27] Dantas-Torres F (2010). Biology and ecology of the brown dog tick, *Rhipicephalus sanguineus*. Parasit. Vectors.

[28] Milanjeet S.H, Singh N.K, Singh N.D, Singh C, Rath S.S (2014). Molecular prevalence and risk factors for the occurrence of canine monocytic ehrlichiosis. Vet. Med.

[29] De Castro M.B, Machado R.Z, De Aquino L.P.C, Alessi A.C, Costa M.T (2004). Experimental acute canine monocytic ehrlichiosis:Clinicopathological and immunopathological findings. Vet. Parasitol.

[30] Villaescusa A, García-Sancho M, Rodríguez-Franco F, Tesouro M.A, Sainz A (2015). Effects of doxycycline on haematology, blood chemistry and peripheral blood lymphocyte subsets of healthy dogs and dogs naturally infected with *Ehrlichia canis*. Vet. J.

[31] Mylonakis M.E, Kritsepi-Konstantinou M, Dumler J.S, Diniz P.P.V, Day M.J, Siarkou V.I, Breitschwerdt E.B, Psychas V, Petanides T, Koutinas A.F (2010). Severe hepatitis associated with acute *Ehrlichia canis* infection in a dog. J. Vet. Intern. Med.

[32] Hildebrandt P.K, Huxsoll D.L, Walker J.S, Nims R.M, Taylor R, Andrews M (1973). Pathology of canine ehrlichiosis. Am. J. Vet. Res.

[33] Codner E.C, Caceci T, Saunders G.K, Smith C.A, Robertson J.L, Martin R.A, Troy G.C (1992). Investigation of glomerular lesions in dogs acute experimentally induced *Ehrlichia canis* infection. Am. J. Vet. Res.

[34] Sarma K, Mondal D.B, Saravanan M (2016). Ultrasonographic changes in dogs naturally infected with tick borne intracellular diseases. J. Parasit. Dis.

[35] Kumar V, Kumar A, Varshney A.C, Tyagi S.P, Kanwar M.S, Sharma S.K (2012). Diagnostic imaging of canine hepatobiliary affections:A review. Vet. Med. Int.

[36] Mylonakis M.E, Koutinas A.F, Billinis C, Leontides L.S, Kontos V, Papadopoulos O, Rallis T, Fytianou A (2003). Evaluation of cytology in the diagnosis of acute canine monocytic ehrlichiosis (*Ehrlichia canis*):A comparison between five methods. Vet. Microbiol.

[37] Silva L.S, Pinho F.A, Prianti M.G, Braga J, Pires L.V, Franca S.A, Silva S.M.M (2016). Renal histopathological changes in dogs naturally infected with *Ehrlichia canis*. Braz. J. Vet. Pathol.

[38] Eddlestone S.M, Diniz P.P, Neer T.M, Gaunt S.D, Corstvet R, Cho D, Hosgood G, Hegarty B, Breitschwerdt E.B (2007). Doxycycline clearance of experimentally induced chronic *Ehrlichia canis* infection in dogs. J. Vet. Intern. Med.

[39] McClure J.C, Crothers M.L, Schaefer J.J, Stanley P.D, Stich R.W (2009). Rapid screening and cultivation of *Ehrlichia canis* from refrigerated carrier blood. Clin. Microbiol. Infect.

